# Anthocyanin Accumulation in Black Kernel Mutant Rice and its Contribution to ROS Detoxification in Response to High Temperature at the Filling Stage

**DOI:** 10.3390/antiox8110510

**Published:** 2019-10-25

**Authors:** Syed Hassan Raza Zaidi, Shamsu Ado Zakari, Qian Zhao, Ali Raza Khan, Jawad Munawar Shah, Fangmin Cheng

**Affiliations:** 1 Institute of Crop Science, College of Agriculture and Biotechnology, Zhejiang University, Hangzhou 310058, China; 11316112@zju.edu.cn (S.H.R.Z.); shamsuado@zju.edu.cn (S.A.Z.); qzh@zju.edu.cn (Q.Z.); alirazakhan.qau@gmail.com (A.R.K.); 2 College of Agriculture, Bahauddin Zakariya University Sub-Campus Bahadur, Layyah 31200, Pakistan; shah8712@yahoo.com

**Keywords:** *Oryza sativa* L., color rice, pigment accumulation, anthocyanin biosynthesis, antioxidant metabolism, high temperature

## Abstract

Effect of high temperature (HT) on anthocyanin (ANS) accumulation and its relationship with reactive oxygen species (ROS) generation in color rice kernel was investigated by using a black kernel mutant (9311*bk*) and its wildtype (WT). 9311*bk* showed strikingly higher ANS content in the kernel than WT. Just like the starch accumulation in rice kernels, ANS accumulation in the 9311*bk* kernel increased progressively along with kernel development, with the highest level of ANS at kernel maturity. HT exposure evidently decreased ANS accumulation in 9311*bk* kernel, but it increased ROS and MDA concentrations. The extent of HT-induced decline in kernel starch accumulation was genotype-dependent, which was much larger for WT than 9311*bk*. Under HT exposure, 9311*bk* had a relatively lower increase in ROS and MDA contents than its WT. This occurrence was just opposite to the genotype-dependent alteration in the activities of antioxidant enzymes (SOD, CAT and APX) in response to HT exposure, suggesting more efficiently ROS detoxification and relatively stronger heat tolerance for 9311*bk* than its WT. Hence, the extent of HT-induced declines in grain weight and kernel starch content was much smaller for 9311*bk* relative to its WT. HT exposure suppressed the transcripts of *OsCHS*, *OsF3’H*, *OsDFR* and *OsANS* and impaired the ANS biosynthesis in rice kernel, which was strongly responsible for HT-induced decline in the accumulation of ANS, C3G, and P3G in 9311*bk* kernels. These results could provide valuable information to cope with global warming and achieving high quality for color rice production.

## 1. Introduction

Rice (*Oryza sativa* L.) is one of the most important cereal crops in the world, especially in Asian countries. Although white rice is the most widely consumed form of rice, there are many special rice cultivars that contain color pigments, such as black, red and purple rice [1]. The kernel color of rice is considered as one of most important traits for the improvement of grain quality, due to its potential biological and pharmacological applications. Previous studies have confirmed that the phenotype of rice kernel is attributed to the accumulation of anthocyanins in pericarps [2]. Anthocyanins are natural colorants belonging to the category of bioflavonoids, which may be divided into two types of chemical compounds: anthocyanins (ANS) and anthocyanidins [3]. Two types of anthocyanins share a common basic core structure, namely the flavylium ion structure [2,3]. In fact, anthocyanidins are the sugar-free or deacyl-glycosylated forms of anthocyanins, while ANS are the glycosylated or acyl-glycosylated forms of anthocyanins by coupling sugar to the anthocyanidin molecule [2,3,4,5]. For two types of anthocyanins, ANS was mostly abundant in cereal kernels that may function as antioxidants [2,3]. In rice, the ANS in color pericarps are comprised of peonidin-glucoside (P3G), cyaniding-3-O-glucoside (C3G) and other-3 glycosides [6]. The amount of different types of ANS in color rice pericarps is greatly variable, depending on the cultivar and growth environment. For instance, the rice cultivars with black kernel pericarps generally have higher P3G and C3G content than those with red kernel [6].

High temperature during grain filling is one of the most important environmental factors affecting crop yield and grain quality [7]. Previously, great efforts have been made to recognize the effect of high temperature on rice grain quality. It has been demonstrated that high temperature at grain filling stage causes the formation of loosely packed starch granules with larger air space, which may result in reduced kernel weight together with increased occurrence of chalky and abnormal grains [6,7]. High temperature (HT) exposure at the grain filling stage obviously reduces the ratio of amylose to total starch accumulation in non-waxy rice endosperms [7,8]. Furthermore, the palatability property of cooking rice cultivars may be remarkably compromised due to the alteration of starch gelatinization resistance in rice endosperms after rice plants exposure to high temperature during grain filling [7,9]. However, little information is available about the impact of high temperature on the expression of key gene related to ANS biosynthesis and the consequent variations in ANS accumulation and its composition in color rice kernels.

The ANS biosynthesis in higher plants was regulated by several key enzymes in the phenylpropanoid pathway, such as isomerase (CHI), chalcone synthase (CHS), flavone 3-hydroxylase (F3H), flavonol synthase (FLS) and flavonoid 3′-hydroxylase (F3′H) [8]. Previous studies have revealed that nutrient deficiencies, especially of phosphorus (P) and nitrogen (N), may induce the accumulation of anthocyanin and anthocyanidin compounds in many plant species [9]. Low temperatures significantly up-regulated the expression of anthocyanin biosynthesis genes, which led to a marked increase in ANS amount in red-skinned grapes [10]. Interestingly, environmental stresses (e.g., chilling, drought and salinity) may cause the oxidative damage of plant cells by rapidly enhancing generation of reactive oxygen species (ROS), like O_2_^−^ (superoxide), H_2_O_2_ (hydrogen peroxide) and OH^−^ (hydroxyl radical) [11]. However, the overexpression of anthocyanin regulatory gene IbMYB1 in potato was found to have relatively higher ANS concentration, lower ROS accumulation and stronger tolerance against chilling adversity than its wild types [12]. Several other studies also have suggested that the over-accumulation of antioxidant flavonoids significantly increases Arabidopsis survival under chilling stress. Hence, the ANS accumulation in plant tissues may function as the antioxidants that protect plant against the oxidative damage from stressful chilling and salinity adversities [13]. By contrast, the benefit of suppressing ANS biosynthesis at high temperatures is unknown. In general, plant cells possess a well-organized antioxidant defense system for detoxification of ROS. The defense system contains both enzymatic antioxidants like catalase (CAT), superoxide dismutase (SOD) and ascorbate peroxidase (APX) and non-enzymatic antioxidants such as anthocyanin, tocopherols, carotenoids and flavonoids [11]. However, it is unclear whether or not the enhanced ANS accumulation in color rice kernels is beneficial for the improvement of anti-oxidative ability of rice under HT exposure.

In this study, two *indica* rice cultivars, 9311 and its corresponding mutant with the black kernel phenotype (9311*bk*), were utilized to compare their differences in pigment content and ANS composition in developing kernels under HT exposure. HT–induced alteration in the expression of various key genes responsible for ANS biosynthesis and its relation to ROS accumulation in developing kernels was further investigated in 9311*bk* and its wild type plants at grain filling stage. The objectives of our research were: 1) to clarify the temporal pattern of kernel ANS accumulation and ANS biosynthesis metabolism in response to HT exposure for color rice and 2) to examine the pleiotropic effects of altered ANS accumulation metabolism on the ROS detoxification in developing kernels of color rice when rice plants was imposed to HT at the grain filling stage.

## 2. Materials and Methods

### 2.1. Plant Materials and Temperature Treatments

**Figure 1:**
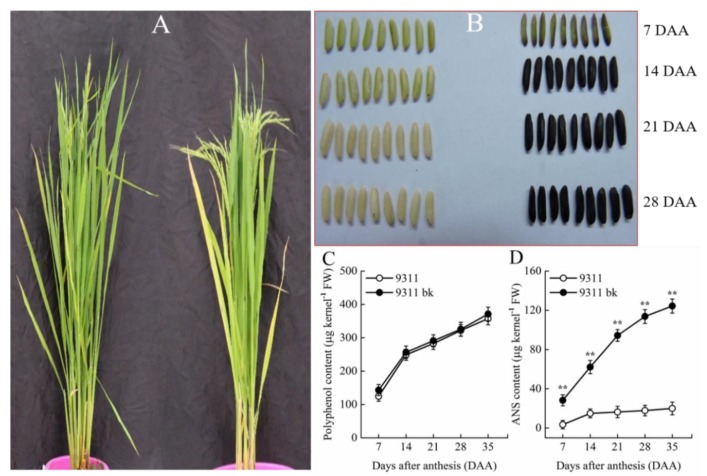
Comparison of plant phenotypes between *bk* mutant (9311*bk*) and its wild type (9311) at heading stage. (**A**) Plant growth phenotype, left: 9311 and right: 9311*bk*. (**B**) Grain appearance (the samples harvested at 7 days interval after anthesis), left: 9311 and right: 9311*bk*. (**C**) Temporal pattern of polyphenol content and (**D**) Temporal pattern of anthocyanin (ANS) content during kernel development. DAA indicates the days after anthesis. Vertical bars represent standard errors (*n* = 3). The asterisks represent significant differences (** *p* < 0.01).

Two rice genotypes, 9311 and its corresponding mutant with the black kernel phenotype, 9311*bk*, were used in the study. The wild type 9311 is a well-known commercial *indica* rice cultivar with its full genome sequence accessible at http://rise2.genomics.org.cn/. The 9311*bk* is derived from 9311 cultivar (*Oryza sativa* L. ssp. indica) by gamma-irradiated 9311 mature seeds, and the stably inherited mutant was acquired through the sequential self-pollination and their identifications of black kernel phenotype in M2–M7 generations, with M8 seeds being employed in this experiment. The extent of black color rice was different among different lines, further characterization of mutation genes will be carried out in future. No visible differences in plant height, panicle morphology, leaf color, grain shape and kernel polyphenol content were observed between the 9311*bk* and its wild type (Figure 1A,C). However, 9311*bk* exhibited a black pericarp (Figure 1B), with the strikingly higher kernel ANS content for 9311*bk* compared with its wild type (Figure 1D).

The experiments of different temperature treatments were performed in 2018 at the experimental station of Zhejiang University, Hangzhou, China. Rice seeds were sown on April 25 and 25–30 days old seedlings were transplanted into the plastic pots filled with air-dried, fully mixed and pre-soaked clay loam soil. The soil contained 1.36 g/kg total N, 24.5 mg/kg available P and 129.3 mg/kg exchangeable K. The pots were placed in a greenhouse under natural light cycles and moderate temperature environment (28 °C day-time/22 °C night-time). At full heading stage, 30–40 panicles with uniform anthesis were randomly selected and tagged. Afterward, the pots were moved to two phytotrons (PGV-36; CONVIRON, Winnipeg, Manitoba, Canada) to impose the different temperature treatments until maturity.

One phytotron was used for high temperature (HT) and the other for normal temperature (NT). The daily mean temperatures were set to 32 °C for HT and 22 °C for NT. The diurnal change of temperature was designed by a simulation of daily temperature fluctuation based on natural climate. The daily maximum and minimum temperatures were set up at 2:00 p.m. and 5:00 a.m., with 36 °C and 28 °C for HT and 24 °C and 20 °C for NT, respectively. Two phytotrons were kept without distinction in other climate conditions except for temperature treatment; the photoperiod was from 5:30 a.m. to 7:00 p.m. with 150 to 180 J m^−2^ s^−1^ of light intensity, and the relative humidity was maintained around 75–80% with a wind speed of 0.5 m s^−1^. After the initiation of temperature treatment (full heading), 3–5 tagged panicles were sampled at 7-day interval up to 28 DAA. The fresh grain samples were frozen in liquid nitrogen and stored at −80 °C until further experimental analysis. Another set of samples was fixed in an oven at 105 °C for 30 min and then dried to constant weight at 80 °C for the determination of sugar and starch.

### 2.2. Determination of Anthocyanin, Flavonoid and Polyphenol Contents in Rice Grains

The ANS concentration in rice grains was measured spectrophotometrically as previously described by Teng, et al. [14]. The ANS amount was calculated as the product of extraction solution volume and relative ANS concentration. One ANS unit equals one absorbance unit (A_530_ − (1/4 × A_657_)) in 1 mL of extraction solution.

Total phenolic content of rice grain was measured according to the method of Dewanto, et al. [15]. Total flavonoid content of rice grain was measured according to the method of Zhishen, et al. [16], with catechin (Sigma, Saint Louis, Missouri, USA) being used as a standard. The total flavonoid contents were expressed as catechin equivalents.

### 2.3. Measurement of ANS Composition by HPLC

The extraction of grain anthocyanin was conducted by using the procedures described previously Yoshimura, et al. [17]. Briefly, 1 g of the ground rice samples was homogenized in 10 mL of methanol containing 1% HCl (*v*/*v*) for 24 h in the dark at 4 °C. The crude extracts were filtered with Whatman No. 2 paper. These crude extracts were then subjected to HPLC.

HPLC analysis was performed using a liquid chromatography system (SCL-10A, Shimadzu, Kyoto, Japan) equipped with two pumps (LC-10A), a control system (SCL-10A), a diode array detector (SPD-20A), a CTA-20A column oven and a column COSMOSIL 5C18-AR-II 4.66250 mm i.e., 5 mm (Nacalaitesque, Kyoto, Japan). The mobile phase consisted of water: formic acid (96:4, *v*/*v*; A phase) and methanol: formic acid (96:4, *v*/*v*; B phase). Sample elution used the time gradient program: 0–0.01 min 10% B, 0.01–5.00 min 20% B; 5.01–50.00 min 50% B and 50.01–60.00 min 100% B. Ten mL of sample was injected. Quantification was performed at l = 520 nm. All chromatographic tests were carried out at 37 °C with a flow of 0.5 mL/min. Cyanidin-3-O-glucoside and peonidin-3-O-glucoside were quantified using a calibration curve of the corresponding standard compounds. All determinations were performed in triplicate.

### 2.4. RNA Isolation, cDNA Preparation, and Real-Time Fluorescence Quantitative PCR

The procedures of RNA extraction and cDNA preparation for rice grains were performed as described previously [8], with some modifications. Trizol Plus reagent kit (Invitrogen, Carlsbad, CA, USA) was used for the extraction of total RNA and the First Strand cDNA Synthesis kit (Toyobo, Osaka, Japan) was used for cDNA synthesis by following the manufacturer’s instructions. Quantitative real-time PCR was performed by using the SYBR Green real-time PCR Master Mix Reagent Kit (Toyobo, Osaka, Japan). The reactions were performed in a Bio-Rad CFX96 real-time system (Bio-Rad, Hercules, CA, USA) by following the manufacturer’s protocol. The amplification reagents contained 10 μL SYBR, 1 μL cDNA, 1.6 μL 10 mM primer pairs and 7.4 μL RNase Free H_2_O. All gene-specific primer pairs were designed by using online software GenScript, and the optimal primer of annealing temperature for each gene was listed in Table S1. The expression of *Actin* was used as internal control. The amplification of various genes was normalized by *ACTIN-1* expression, and their relative expression levels were calculated by the 2^(−ΔΔCT)^ method [18]. The average values and standard errors were calculated from three independent biological replicates.

### 2.5. Determination of Malondialdehyde (MDA), Hydrogen Peroxide (H_2_O_2_), Superoxide Radical (O_2_^−^) and Antioxidant Enzyme Activity

The MDA and O_2_^−^ contents in rice grains were determined spectrophotometrically as described by Lichtenthaler [19]. The H_2_O_2_ concentration was measured calorimetrically as described by Khan et al. [20].

SOD and APX activities were assayed, according to Cakmak and Marschner [21]. CAT activities were measured as described by Khan et al. [20]. Data collections of enzyme activities were carried out on a Shimadzu UV-vis 2450/2550 spectrophotometer (Shimadzu, Kyoto, Japan). Triplicate measurements were assayed for each sample.

### 2.6. Determination of Carbohydrates

The extraction of carbohydrate was performed by following the method described by Li et al. [22].The samples were dried in an oven and ground into fine powder. 500 mg ground sample was added in a 10 mL centrifuge tube, containing 5 mL of 80% (*v*/*v*) ethanol and kept in a water bath at 80 °C for 30 min. The tube was then centrifuged at 5000× *g* for 15 min after cooling. The extraction process was repeated three times. The supernatant was then diluted to 25 mL with distilled water and soluble sugar content was measured as described by Yang, et al. [23]. The residue in the centrifuge tube was dried at 80 °C for starch extraction. 2 mL distilled water was added to the tube, which was then shaken in a boiling water bath for 15 min, followed by addition of 2.5 mL of 9.36 M HClO_4_ added after cooling. The solution was shaken for 15 min and centrifuged at 5000× *g* for 15 min. The supernatant was collected and a 2 mL of 4.68 M HClO_4_ was added to the residue. The extraction was repeated as mentioned above. The supernatants were combined and diluted to 25 mL with distilled water. The starch content was measured by the method of Yang et al. [23].Triplicate measurements were assayed for each sample.

### 2.7. Statistical Analysis

The data were subjected to a two-way analysis of variance (ANOVA) using the IBM SPSS statistical software 20 package (Chicago, IL, USA). The means were compared by the Tukey’s least significant difference (LSD) test (*p* < 0.05). Standard error (SE) was computed and illustrated in the figures and tables.

## 3. Results

### 3.1. Differences in Kernel Pigment Concentration and ANS Composition Between 9311bk and Wild Type under Different Temperature Regimes

**Figure 2:**
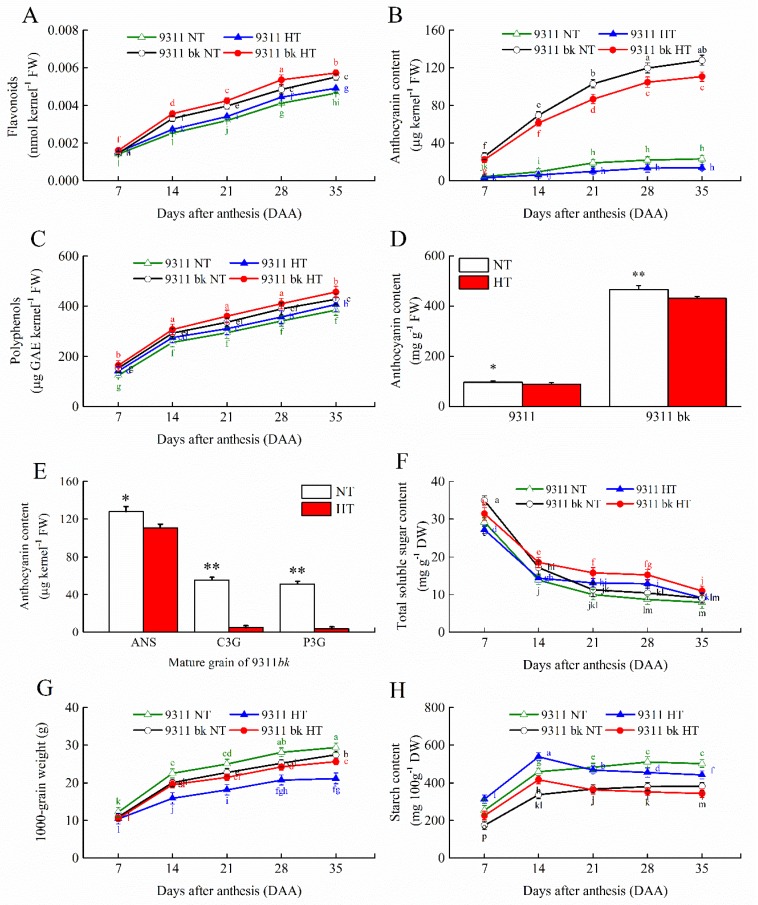
Effect of high temperature on pigment accumulation in the rice kernel. (**A**) Temporal pattern of flavonoid content in kernel of 9311 and 9311*bk*, (**B**) temporal pattern of anthocyanin content in kernel of 9311 and 9311*bk*, (**C**) temporal pattern of polyphenol content in kernel of 9311 and 9311*bk*, (**D**) anthocyanin content in mature grains of 9311 and 9311*bk*, (**E**) anthocyanin (ANS), cyanidin-3-O-glucoside (C3G) and peonidin-3-O-glucoside (P3G) content in mature grain of 9311*bk* measured by HPLC, (**F**) temporal pattern of total soluble sugar content in kernel of 9311 and 9311*bk,* (**G**) temporal pattern of 1000-grain weight of 9311 and 9311*bk* and (**H**) temporal pattern of starch content in kernel of 9311 and 931*1bk* after anthesis. Vertical bars represent standard errors (*n* = 3). Different letters represent significant differences (*p* < 0.05) and the asterisks also represent significant differences (* *p* < 0.05 and ** *p* < 0.01). DAA stands for days after anthesis.

9311*bk* differed evidently from WT in flavonoid and ANS contents during grain filling periods (7–35 DAA), with the strikingly higher ANS content for 9311*bk* relative to WT under the same temperature treatment (Figure 2A,B). The kernel flavonoid and ANS contents increased progressively along with grain filling, with the largest amount in mature kernels (Figure 2A,B). On the other hand, the insignificant difference in polyphenol content was found between 9311*bk* and WT (Figure 2C). These results clearly indicated that the black color of 9311*bk* kernels was chemically derived from the ANS accumulation, rather than the polyphenol and other pigment.

As shown in Figure 2D, HT exposure at grain filling significantly decreased the total ANS content (mg/g) in 9311*bk*, and HT-induced decline in kernel ANS content was also shown for WT (Figure 2D). Furthermore, HT exposure significantly decreased the accumulation of ANS, C3G and P3G in 9311*bk* kernels on a per-kernel basis (μg kernel^−1^; Figure 2E), and the gap in kernel ANS concentration between two temperature regimes widened progressively with the advancement in grain development (Figure 2D,E). Interestingly, the effect of HT exposure on grain weight and kernel starch accumulation (Figure 2H,G) was quite different from that on total ANS, C3G and P3G concentrations in rice kernels (Figure 2D,E). For instance, the starch content and grain weight in HT-ripening kernel were significantly lower than those of NT-ripening kernel at maturity, although HT exposure accelerated grain filling and enhanced the starch concentration at the early stage of grain filling (Figure 2H,G). Comparatively, the extent of HT-induced decline in kernel starch content was relatively smaller for 9311*bk* than WT at later stage of grain filling (Figure 2H). The soluble sugar content in 9311*bk* kernels was relatively higher than WT kernels under the same temperature regime (Figure 2F), and HT-inducible increase in total soluble sugar contents was found for both 9311*bk* and WT (14–28 d post anthesis; Figure 2F).

### 3.2. Expression Pattern of Various Genes Involved in ANS Biosynthesis in Developing Kernels and Its Response to HT Exposure at Grain Filling

**Figure 3:**
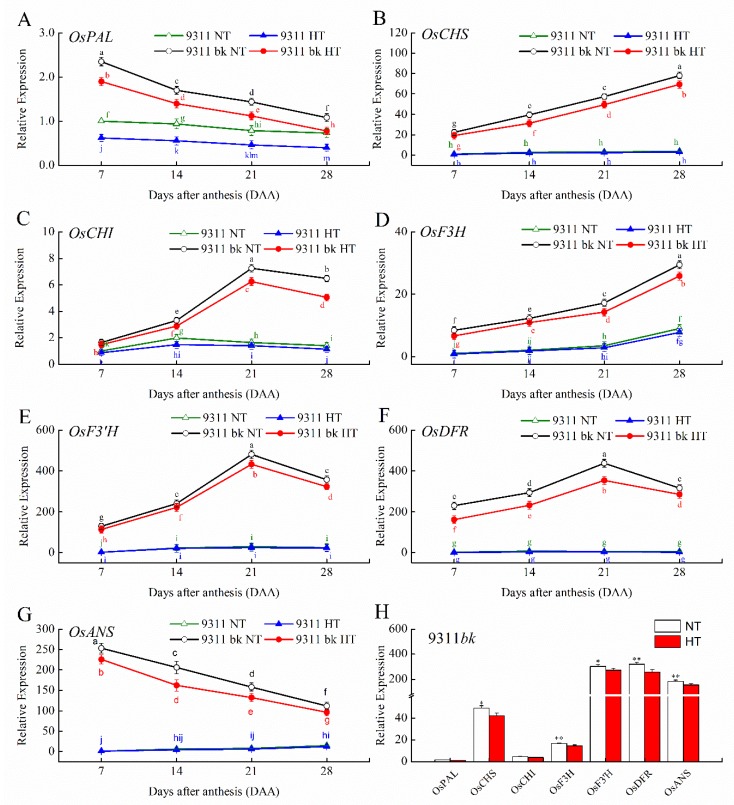
Effect of the high temperature on the expression of anthocyanin biosynthesis genes and their temporal patterns in 9311*bk* and WT kernels. (**A**–**G**) The time course of *OsPAL, OsCHS*, *OsCHI*, *OsF3H*, *OsF3’H*, *OsANS* and *OsDFR* expression during kernel development and (**H**) comparison of relative expression of genes involved in anthocyanin biosynthesis in developing kernel of 9311*bk* at after anthesis. Vertical bars represent standard errors (*n* = 3). Different letters represent significant differences (*p* < 0.05) and the asterisks also represent significant differences (* *p* < 0.05 and ** *p* < 0.01). DAA stands for days after anthesis.

The transcriptional profile of various genes involved in ANS biosynthesis pathway (*OsPAL*, *OsCHS*, *OsCHI*, *OsF3H*, *OsF3’H*, *OsDFR* and *OsANS*) and also their temporal patterns during developing kernels were comprehensively investigated by using quantitative real-time reverse transcription PCR (Figure 3). The result showed that 9311*bk* had the strikingly higher transcripts of *OsCHS*, *OsCHI*, *OsF3H*, *OsF3’H*, *OsDFR* and *OsANS* in developing kernels than its wild type. The *OsPAL* transcripts in the developing kernels of 9311*bk* also showed significantly higher expression than those of WT at the earlier filling stage (7 d and 14 d post anthesis). Obviously, the transcripts of *OsCHS*, *OsF3’H*, *OsDFR* and *OsANS* in WT kernels were detectable at very low levels (Figure 3B,E–G) except *OsPAL,* which was relatively abundant in WT kernels (Figure 3A). For the temporal pattern of transcriptional expression of various genes in 9311*bk* kernels, the transcripts of *OsCHS* and *OsF3H* decreased gradually along with grain filling (Figure 3B,D), but the opposite was true for *OsANS*, which exhibited an abrupt decline in transcript level along with grain filling (Figure 3F). In addition, the maximum values of *OsCHI*, *OsF3’H* and *OsDFR* were observed at the middle and late stage (21 d post anthesis; Figure 3C,E,F). Interestingly, the transcript levels of all the seven genes (*OsPAL*, *OsCHS*, *OsCHI*, *OsF3H*, *OsF3’H*, *OsDFR* and *OsANS*) in 9311*bk* kernels were strongly repressed by HT exposure at grain filling stage, in relation to the NT treatment at the same sampling time (Figure 3). This result suggested that HT exposure had an inhibitory impact on the ANS biosynthesis in rice kernel, which was strongly responsible for HT-induced decline in the accumulation amounts of ANS, C3G and P3G in 9311*bk* kernels.

### 3.3. Difference in the Response of Kernel ROS Content, MDA Accumulation and Antioxidant Enzyme to HT Exposure Between 9311bk and Its Wild Type

**Figure 4:**
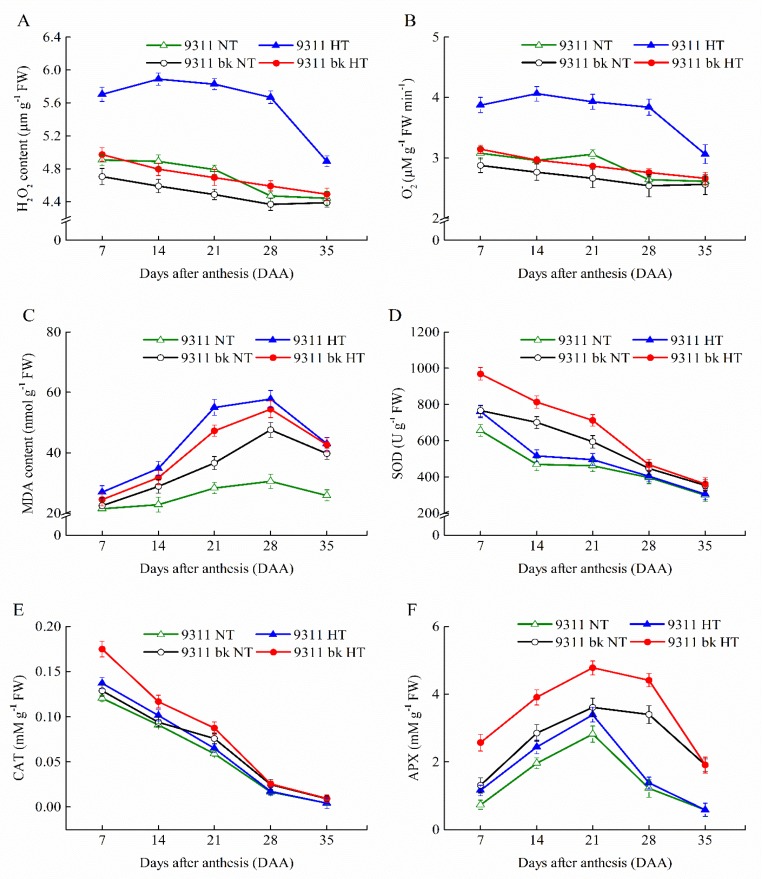
Effect of high temperature on reactive oxygen species (ROS) content and antioxidant enzyme activity in 9311 and *9311bk* during kernel filling process towards maturity. (**A**) H_2_O_2_ content, (**B**) O_2_^−^ content, (**C**) MDA content, (**D**) SOD activity, (**E**) CAT activity and (**F**) APX activity. Vertical bars represent standard errors (*n* = 3). DAA stands for days after anthesis.

The genotype-dependent alterations in the kernel ROS level, MDA content and the activities of antioxidant enzymes, in response to HT exposure, were investigated in 9311*bk* and WT (Figure 4). The results showed that HT exposure led to the marked increases in ROS level (H_2_O_2_ and O_2_^−^ contents) and MDA content in developing kernels for both genotypes (Figure 4A–C). However, the extent of HT-induced increase in kernel ROS level and MDA content was greatly variable between the rice genotypes (9311*bk* and WT). The elevated extent of ROS level and MDA content in 9311*bk* kernels was evidently smaller than those in WT under HT exposure (Figure 4A–C). This result implied that 9311*bk* was less susceptible to HT exposure and also depicted relatively lower oxidative damage under HT exposure than WT. Interestingly, the developing kernels of two genotypes significantly differed in terms of antioxidant enzyme activities. Relatively higher activities of SOD, CAT and APX were observed for 9311*bk*, as compared to WT, under exposure to HT (Figure 4D–F).

## 4. Discussion

**Figure 5:**
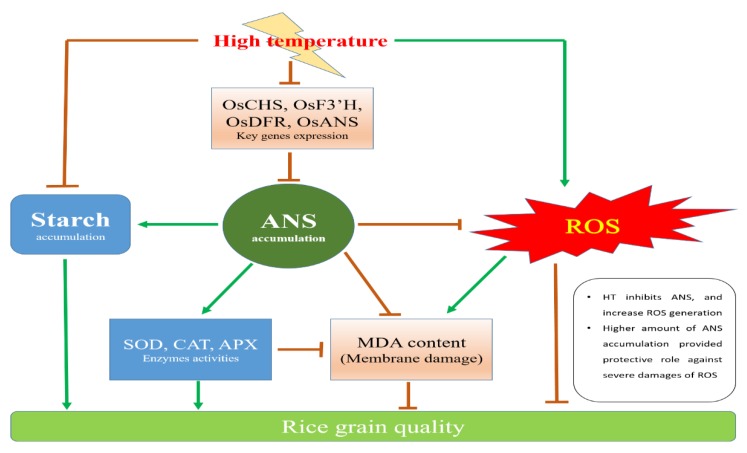
Schematic representation about the involvement of ANS accumulation to ameliorate high temperature-induced oxidative damage in filling kernel of rice.

Anthocyanin biosynthesis is an energy-demanding process and energy balance might be optimized in response to the varying environment. ANS biosynthesis is increased at cold temperatures, and this increase has been suggested to enhance the survival of plants under cold stress [24]. In the current study, HT exposure suppressed the ANS accumulation in rice kernels (Figure 2B) and inhibited the transcriptional expression of various genes involving in the ANS biosynthesis pathway (Figure 3). However, the ANS content in 9311*bk* kernels under HT exposure still was strikingly higher than its WT. Previous studies had revealed that HT exposure significantly enhanced the rate of grain filling, but it decreased the grain weight and final yield [25,26,27]. Our present data provided the supporting evidence that HT exposure at the filling stage significantly lowered grain weight and starch amount accumulation in rice kernels. However, the extent of HT-induced declines in grain weight and kernel starch content was relatively smaller for 9311*bk* than WT (Figure 2H,G), implying that the starch accumulation in 9311bk was less affected by HT exposure than in wild type (Figure 2H). Interestingly, 9311*bk* had significantly lower ROS concentration and higher activity of antioxidant enzymes (SOD, CAT and APX) in developing kernels than its WT under HT exposure (Figure 4). Hence, we deduced that ANS rich 9311*bk* has a stronger defense ability to scavenge ROS molecules efficiently than its WT, and the genotype-dependent alterations in ANS biosynthesis and its accumulation in color kernels might contribute greatly to the enhancements of kernel-filling capacity and its heat tolerance under HT stress (Figure 5). The possible reason for the elevated heat tolerance of color rice could be explained by the combination of two factors: Firstly, ANS biosynthesis and its accumulation in plant tissues may play an important role in protecting plant against ROS damage, because of ANS being widely considered as antioxidant molecules in plant cells [28]. In previous studies, Gould, et al. [29] revealed that anthocyanin-rich parts of *Pseudowintera colorata* leaves showed higher H_2_O_2_ scavenging ability as compared to their green counterparts when plant leaves were exposed to mechanical injury. Cotton plants, suffering from nutrient imbalance, were reported to accumulate higher anthocyanins in leaves under drought stress with a consequent higher ROS-scavenging ability [30]. In our present study, HT exposure enhanced ROS concentration and MDA accumulation in rice kernels (Figure 4A–C), which was well consistent with previous reports [31]. However, 9311*bk* differed evidently from its wildtype in the response of MDA accumulation to HT exposure (Figure 4C), and the concentrations of H_2_O_2_ and O_2_^−^ in 9311*bk* kernels were significantly lower than those in its wildtype kernels under HT exposure (Figure 4A,B). More importantly, the kernel flavonoid and ANS contents in 9311*bk* kernels increased progressively along with grain filling, with the largest amount in its mature kernels (Figure 2A,B), while the ANS content in WT kernels was detectable at a very low level (Figure 2A,B). In this regard, ANS accumulation might be directly responsible for the alleviation of oxidative damage in color rice kernels, due to the striking difference in kernel ANS accumulation amount between 9311*bk* and its WT (Figure 2A,B); secondly, the altering ANS biosynthesis in color rice kernels may also play a regulatory role in the activation of the antioxidant enzyme system, because the antioxidant enzymes (SOD, CAT and APX) had been widely considered as an important mechanism actively used by plants to detoxify ROS damage [20,32]. Several previous studies had also revealed that the ANS accumulation in plant tissues was closely associated with the activities of SOD and CAT for better antioxidant defense system under stressful environment [33,34,35,36]. Thus, the ANS accumulation in plant tissues may also stimulate the activation of the antioxidant enzyme system, this occurrence was another important reason for the elevated heat tolerance of color rice and the relatively stable starch accumulation in 9311*bk* kernels under the HT exposure at filling stage.

The production of ANS was markedly increased under various environmental stresses [24,31]. ANS are believed to be regulated by sugar [28], and light [37]. Sugars activated the accumulation of anthocyanins in numerous plant species like *Arabidopsis*, petunia, grape and radish (*Raphanus sativus* L.) hypocotyls [28,38,39,40]. Sugar is a common regulator of gene expression for metabolic enzymes and proteins involved in photosynthesis, carbohydrate metabolism and ANS biosynthesis [41]. Previous studies have investigated the sugar-induced accumulation of ANS in fruits [42,43]. *CHS* was induced by sugars in transgenic *Arabidopsis* leaves and the genes, encoding *DFR* and *ANS,* were found to be up-regulated under chilling stress [42,44]. Higher light intensities significantly stimulate *CHS* and *CHI* transcript levels in grape (*Vitis vinifera*) cells while *DFR* and *ANS* were not sufficiently stimulated. *Petunia corollas*, cultured in vitro without sucrose, were devoid of pigmentation [45]. A study on *Arabidopsis* revealed that the down-stream genes in the flavonoid biosynthetic pathway were easily induced by sucrose, with several hundred-fold upregulation for such gene transcript under higher sucrose concentration, whereas the transcriptional expression of some genes upstream of *DFR* was relatively stable or only slight up-regulation by sucrose induction [45]. In this study, 9311*bk* had relatively higher content of total soluble sugar content (TSS) than its WT under the same temperature regime (Figure 2F), which was well consistent with the genotypic-dependent alteration in the ANS accumulation in developing kernels (Figure 2B). Furthermore, HT exposure increased the TSS content in developing kernels at the middle and late stages of grain filling, with more profoundly enhanced TSS content for 9311*bk* relative to it WT (Figure 2F). This result suggested that the genotypic-dependent alteration in the ANS accumulation in developing kernels had a marked impact on kernel TSS content and starch accumulation in response to HT exposure. Interestingly, the TSS content in developing kernels showed decreasing trend during grain filling, while it was opposite for the temporal pattern of kernel ANS accumulation during grain filling, although the TSS content in 9311*bk* kernels was significantly higher than its WT (Figure 2B). This phenomenon could be explained by the translocation from sugar to starch via starch biosynthetic metabolism in rice kernels, because sugar provides substrates for biosynthesis of biopolymers such as starch, cellulose, callose, protein and ANS in plant tissues [45]. Hence, HT induced-enhancement in kernel TSS content was closely associated with HT-induced decline in ANS accumulation for 9311*bk*. In this aspect, the suppression of anthocyanin biosynthesis might be beneficial for plants under HT stress, because thermomorphogeneic growth, at a cost of reduced anthocyanin biosynthesis, might enhance survival under HT stress [31]. Obviously, HT exposure suppressed the transcript amounts of various genes involving in ANS biosynthesis in developing kernels (Figure 3), despites the different expression in their temporal pattern during kernel development. For instance, the transcripts of *OsCHS*, *OsANS* and *OsF3H* dropped continuously along with kernel development (Figure 3B,D,G), while the transcripts of *OsCHI*, *OsF3’H* and *OsDFR* evidently enhanced at the early and middle stages, followed by the significantly lowered transcripts at the late filling stages (Figure 3C,E,F). Obviously, the ANS accumulation in 9311*bk* kernels was in parallel to the transcriptional expression of *OsCHS*, *OsCHI*, *OsF3’H* and *OsDFR* during kernel filling under HT exposure, while in wild type the expressions of all the genes were quite low except *OsPAL*. These results implied that such genes (*OsCHS*, *OsCHI*, *OsF3’H* and *OsDFR*) might be the key regulatory genes responsible for the response of ANS biosynthesis in 9311*bk* kernels to HT exposure. The higher expression of *OsPAL* transcript in the developing kernels of both wild type and 9311*bk* (Figure 3A) could be explained by the fact that *PAL* participate in the biosynthesis of various phenolic compounds by phenylpropanoid metabolic pathway [46], besides its involvement in the ANS biosynthesis pathway. In addition, our present result also revealed that the ANS content in color rice kernels increased progressively along with grain filling, with the largest amount in kernels (Figure 2A,B), which also was not well consistent with the temporal pattern of *OsCHS*, *OsCHI*, *OsF3’H* and *OsDFR* during grain filling (Figure 3C,E,F). Considering the relatively slower increases in grain weight and starch accumulation amount at the late stage of grain filling (Figure 2G,H). This discrepancy possibly was attributable to the varying ratio of ANS content to the total amount of starch accumulation and water content in developing kernels, although HT exposure evidently suppressed the accumulation of both ANS and starch in rice kernels (Figure 2A,B,H). Further investigations should be required to clarify the regulatory network for the relationship of ANS metabolism with starch biosynthesis and oxidative homeostasis in color rice kernels as effected by HT exposure at the filling stage.

## 5. Conclusions

HT exposure evidently suppressed the transcriptional expression of various key genes involving in ANS biosynthesis pathway and caused the ANS accumulation amount in color rice kernels. However, the transcripts of various key genes involving in ANS biosynthesis metabolism and ANS concentration in 9311*bk* kernels under HT exposure still was strikingly higher than its WT. Under HT exposure, ANS rich 9311*bk* had relatively lower increase in ROS and MDA contents than its WT, while it was just opposite to the genotype-dependent alteration in the activities of antioxidant enzymes (SOD, CAT and APX) in response to HT exposure, suggesting that 9311*bk* has stronger defense ability to efficiently scavenge ROS than its WT. Hence, the starch accumulation in 9311bk kernel was less affected by HT exposure than in the wild type.

## Supplementary Materials

The following are available online at https://www.mdpi.com/2076-3921/8/11/510/s1, Table S1: List of primers used for anthocyanin biosynthesis genes in this study. Figure S1: HPLC chromatogram of anthocyanidins in 9311*bk* extract.

## Abbreviations

ANS	Anthocyanidins
ROS	Reactive oxygen species
SOD	Superoxide dismutase
POD	Peroxidase
APX	Ascorbate peroxidase
CAT	Catalase
DAA	Days after anthesis
O_2_^−^	Superoxide anion
MDA	Malondialdehyde
Chl	Chlorophyll
