# Supplementary material for: Anthocyanin Accumulation in Black Kernel Mutant Rice and its Contribution to ROS Detoxification in Response to High Temperature at the Filling Stage
Source: Antioxidants (Basel). 2019 Oct 25;8(11):510. doi: 10.3390/antiox8110510 (PMC6912731; doi:10.3390/antiox8110510)

Anthocyanin accumulation in black kernel mutant rice and its contribution to ROS detoxification in response to high temperature at filling stage

Syed Hassan Raza Zaidi[1], Shamsu Ado Zakari[1], Qian Zhao[1], Ali Raza Khan[1], Jawad Munawar Shah[2], Fangmin Cheng[1*].

[1] Institute of Crop Science, College of Agriculture and Biotechnology, Zhejiang University, Hangzhou, 310058, China

[2] College of Agriculture, Bahauddin Zakariya University Sub-Campus Bahadur, Layyah, 31200, Pakistan

*Correspondence, Fangmin Cheng, Institute of Crop Science, Zhejiang University, Hangzhou, 310058, China

Email: chengfm@zju.edu.cn

Telephone: +86 571 86771117

Supplementary tables and figures

Table S1 Sequence of primers for genes involved in Anthocyanin Biosynthesis used for real-time quantitative polymerase chain reaction

Gene	Accession No.	Primer Pairs	Products (bp)
ACTIN	X16280	5’-CAGCACATTCCAGCAGATGT-3’	198
		5’-TAGGCCGGTTGAAAACTTTG-3’	
OsPAL	X16099	5’-GAACATCAAGAGCTCCGTCA-3’	111
		5’-AGGAGGTTCTTCTCGCTGAA-3’	
OsCHS	X89859	5’-GCCGACTACCCGGACTACTA-3’	103
		5’-TCTTCCTGATCTGCGACTTG-3’	
OsCHI	AF474922	5’-TTCTACTGCCCTCGTGTTTG-3’	79
		5’-CAATTCAGAGATGGAACGGA-3’	
OsF3H	AC092697	5’-AGAAGCTCATCACCGACGAC-3’	80
		5’-CAGTGCTCCTGGTCAAGGTT-3’	
OsF3’H	HQ876708	5’-GCGCTACCCTCCTAGTCAAC-3’	88
		5’-AGAAACCGAGAAGGCTGGTA-3’	
OsDFR	Y07956	5’-CGAGAAGGAACCGATACTGG-3’	101
		5’-ACCGTTCTCTGGACTCACTA-3’	
OsANS	Y07955	5’-ACGCAAGCTGTTCAAGAAGC-3’	70
		5’-TATCATTCCGTTCGATGCAG-3’	

Figure S1 HPLC chromatogram of anthocyanidins in 9311*bk* extract;

peak 1, Cyanidin-3-o-glucoside;

peak 2, Peonidin-3-O-glucoside.

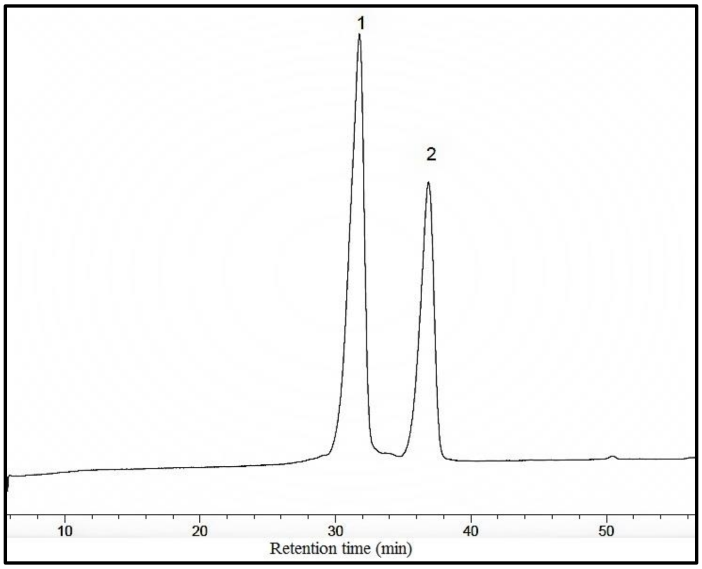

Supplement: Supplementary file 1 [file antioxidants-08-00510-s001.pdf]
